# Norartocarpetin from a folk medicine Artocarpus communis plays a melanogenesis inhibitor without cytotoxicity in B16F10 cell and skin irritation in mice

**DOI:** 10.1186/1472-6882-13-348

**Published:** 2013-12-10

**Authors:** Horng-Huey Ko, Yi-Ting Tsai, Ming-Hong Yen, Chun-Ching Lin, Chan-Jung Liang, Tsung-Han Yang, Chiang-Wen Lee, Feng-Lin Yen

**Affiliations:** 1 Department of Fragrance and Cosmetic Science, College of Pharmacy, Kaohsiung Medical University, Kaohsiung, Taiwan; 2 Graduate Institute of Natural Products, College of Pharmacy, Kaohsiung Medical University, Kaohsiung, Taiwan; 3 School of Pharmacy, College of Pharmacy, Kaohsiung Medical University, Kaohsiung, Taiwan; 4 Department of Anatomy and Cell Biology, College of Medicine, National Taiwan University, Taipei, Taiwan; 5 Laboratory Medicine, Chang Gung Memorial Hospital, Chiayi, Taiwan; 6 Department of Nursing, Division of Basic Medical Sciences, and Chronic Diseases and Health Promotion Research Center, Chang Gung Institute of Technology, Chia-Yi, Taiwan

**Keywords:** Norartocarpetin, Melanogenesis, Tyrosinase, Microphthalmia-associated transcription factor, Mitogen-activated protein kinases

## Abstract

**Background:**

Many natural products used in preventive medicine have also been developed as cosmeceutical ingredients in skin care products, such as *Scutellaria baicalensis* and *Gardenia jasminoides*. Norartocarpetin is one of the antioxidant and antityrosinase activity compound in *Artocarpus communis*; however, the cytotoxicity, skin irritation and antimelanogenesis mechanisms of norartocarpetin have not been investigated yet.

**Methods:**

In the present study, cell viability *in vitro* and skin irritation *in vivo* are used to determine the safety of norartocarpetin. The melanogenesis inhibition of norartocarpetin was determined by cellular melanin content and tyrosinase in B16F10 melanoma cell. Moreover, we examined the related-melanogenesis protein by western blot analysis for elucidating the antimelanogenesis mechanism of norartocarpin.

**Results:**

The result of the present study demonstrated that norartocarpetin not only present non-cytotoxic in B16F10 and human fibroblast cells but also non-skin irritation in mice. Moreover, our result also first found that norartocarpetin downregulated phospho-cAMP response element-binding (phospho-CREB) and microphthalmia-associated transcription factor (MITF) expression, which in turn decreased both synthesis of tyrosinases (TRP-1 and TRP-2) and cellular melanin content. This process is dependent on norartocarpetin phosphorylation by mitogen-activated protein kinases such as phospho-JNK and phospho-p38, and it results in decreased melanogenesis.

**Conclusion:**

The present study suggests that norartocarpetin could be used as a whitening agent in medicine and/or cosmetic industry and need further clinical study.

## Background

**Figure 1:**
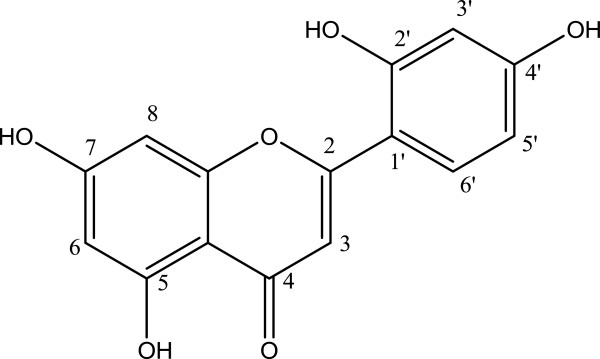
Chemical structure of norartocarpetin.

In recent years, natural products and medicines have been developed as cosmeceutical ingredients to resolve esthetic skin problems such as skin darkening and wrinkle [1-4]. *Artocarpus* species, including *A. heterophyllus*, *A. lakoocha*, *A. communis*, are Asian or Pacific tree crops that are commonly used in agriculture, traditional medicine, and industry [5]. *Artocarpus* species have been shown to possess many pharmacological properties, which include anti-inflammatory [6,7], tyrosinase inhibitory [8], antitumorigenic [9], antidiabetic [10], antibacterial [11], antitubercular [12], antiviral [13], antiplatelet [14], and antioxidant activity [15]. Some of these effects might be due to the antioxidant and anti-inflammatory activity of norartocarpetin (2-(2,4-dihydroxyphenyl)-5,7- dihydroxy-4H-chromen-4- one; Figure 1), a flavonoid compound present in *A. communis* and *A. heterophyllus*[7]. However, the biological pathways that norartocarpetin targets have not yet been fully investigated.

Normal melanin production is required to prevent ultraviolet (UV)-induced DNA damage since it absorbs UV radiation and lowers the occurrence of skin cancer [16,17]. Unfortunately, skin color darkens as a result of excessive exposure to UV radiation due to activation of the alpha-melanocyte stimulating hormone (α-MSH) pathway, which results in melanogenesis [18]. α-MSH, a cyclic adenine monophosphate (cAMP) elevating agent, is usually used to induce the phosphorylation of cAMP response element-binding protein (CREB) and enhance microphthalmia-associated transcription factor (MITF) protein levels [19]. Previous studies have demonstrated that MITF is the major regulator for synthesized tyrosinase (TYR) and its related proteins (TRP-1 and TRP-2) [20,21]. These tyrosinase-related proteins are the rate-limiting enzyme of melanogenesis since they regulate conversion of tyrosine to dopaquinone, rearrangement of DOPAchrome to 5,6-dihydroxy-indole-2-carboxylic acid, and abnormal accumulation of melanin pigments. In addition, phosphorylation of mitogen-activated protein kinases (MAPK) and signaling cascades of extracellular responsive kinase (ERK), c-Jun N-terminal kinase (JNK), and p38 also modulate melanogenesis [22,23].

Therefore, skin whitening agents can inhibit MITF transcriptional activity by decreasing TYR protein levels through MAPK-mediated MITF phosphorylation. The MAPK-mediated MITF degradation pathways activated by norartocarpetin have not been investigated yet. The aim of this study was first to determine the toxicity of norartocarpetin *in vitro* and *in vivo* model and then to define the pathway by which norartocarpetin inhibits the melanogenesis signaling cascade by examining the activation of MITF transcription regulators (p-CREB, MITF, TYR, TRP-1, and TRP-2) and phosphorylation of MAPK signaling pathways (p-ERK, p-JNK, and p-p38).

## Methods

### Chemicals and reagents

Dimethyl sulfoxide (DMSO), α-MSH, 3-(4,5-dimethyl-thiazol-2-yl)-2, 5-diphenyl tetrazolium bromide (MTT), and l-DOPA were purchased from Sigma-Aldrich Chemicals Co. (St. Louis, MO, USA). U0126, SB202190, SP600125, were from Biomol (Plymouth Meeting, PA, USA). phospho-ERK (p-ERK) (Thr202/Tyr204), p-p38 (Thr180/Tyr182), p-JNK (Thr183/Tyr185), and p-CREB (Ser 133) antibodies were purchased from Cell Signaling Technology (USA). MITF, TYR, TRP1, TRP-2, GAPDH, anti-mouse, anti-goat, and anti-rabbit IgG antibodies (horseradish peroxidase conjugated) were bought from Santa Cruz Biotechnology (USA). U0126 (selective inhibitor of MAPK/ERK), SB202190 (selective inhibitor of p38), and SP600125 (selective inhibitor of JNK) were purchased from Biomol (Plymouth Meeting).

### Norartocarpetin purification

The heartwood of *A. communis* was obtained from Tainan district agricultural research and extension station, Council of Agriculture, Taiwan. The plant species was authenticated by Dr. Ming-Hong Yen of the Graduate Institute of Natural Products, College of Pharmacy, Kaohsiung Medical University, Kaohsiung, Taiwan. The voucher specimen of *A. communis* J.R. Forst. & G. Forst (2001-ACHW) has been deposited at the Herbarium of the Department of Fragrance and Cosmetic Science, Kaohsiung Medical University, Kaohsiung, Taiwan. Two kilograms of *A. communis* heartwood was sliced and immersed in a glass container containing methanol at room temperature. This procedure was repeated 3 times. The methanol extract was blended and concentrated using rotary vacuum evaporation. The dried extract (160 grams) was then dissolved with equal volume of dichloromethane (DCM) and ethyl acetate (EA). The EA partition was subjected to silica gel column chromatography and eluted with different proportions of *n*-hexane/EA (19:1 and 8:2) collected solution was then eluted with an equal proportion of DCM/EA and DCM/acetone (2:1). The fraction was then purified on a Sephadex LH-20 column to obtain norartocarpetin. Norartocarpetin is a light yellow powder. The UV spectrum of norartocarpetin in methanol showed absorption maxima at 263 and 350 nm. The IR spectrum showed hydroxyl, conjugated carbonyl and aromatic ring absorption bands at 3071, 1661 and 1619 cm^-1^, respectively. The electrospray ionization mass spectrometry (ESIMS) of norartocarpetin gave a [M + H]^+^ peak at m/z 287 and a [M + Na]^+^ peak at m/z 309, which corresponded to a molecular formula of C_15_H_10_O_6_. The structure of norartocarpetin (Figure 1) was also determined using NMR. The NMR data is as follows: ^1^H-NMR (400 MHz, Acetone-*d*_6_): *δ* 6.19 (1H, d, *J* = 2.4 Hz, H-6), 6.42 (1H, d, *J* = 2.4 Hz, H-8), 6.42 (1H, d, *J* = 2.4 Hz, H-3′), 6.60 (1H, dd, *J* = 8.8, 2.4 Hz, H-5′), 7.15 (1H, s, H-3), 7.79 (1H, d, *J* = 8.8 Hz, H-6′), 13.14 (1H, s, OH-5); ^13^C-NMR (100 MHz, Acetone-*d*_6_): *δ* 94.8 (C-8), 99.8 (C-6), 104.1 (C-3′), 105.1 (C-4a), 108.3, (C-3), 109.1 (C-5′), 110.7 (C-1′), 131.0 (C-6′), 159.4 (C-2′), 160.4 (C-4′), 163.1 (C-5), 163.3 (C-8a), 164.2 (C-7), 165.8 (C-2), 184.4 (C-4). Norartocarpetin was collected and stored in a moisture-proof container until further use.

### Cytotoxicity of norartocarpetin

B16F10 melanoma cells and human fibroblast cells (Hs68 cell line) were purchased from BCRC (Bioresource Collection and Research Center, Hsinchu, Taiwan), which originally purchased them from ATCC (USA). B16F10 melanoma cells were cultured in complete DMEM (Life Technologies, USA) (10% fetal bovine serum, 100 units/ml penicillin G, 100 μg/ml streptomycin, and 0.25 μg/ml amphotericin B) in an incubator at 37°C with 5% CO_2_. Briefly, 1 × 10^4^ B16F10 cells and human fibroblast cells were seeded in 96-well culture plates and allowed to adhere for 24 h. After adhesion, a series of norartocarpetin concentrations were dissolved in DMSO, diluted in DMEM medium, and added into each well for 48 h. At the end of the incubation, the residual medium was removed, and 150 μl of 5 mg/ml MTT solution was added to each well and incubated for 4 h at 37°C. The medium was removed, and 100 μl DMSO was added to each well, which was then gently shaken. The 96-well plates were then quickly measured at 550 nm with a microplate spectrophotometer (BIOTEK, μQuant, USA). The absorbance of cells treated with DMSO was considered the control and compared with that at different norartocarpetin concentrations. All determinations were performed in triplicate.

### Skin irritation of norartocarpetin

The evaluation of skin irritation is the major index of dermal safety in cosmetic application and therefore the dermal safety of norartocarpetin was conducted according to the Draize test described by ISO-10993-10 (Tests for irritation and skin sensitization. The protocol has been reviewed and approved by the Institutional Animal Care and Use Committee (IACUC) of Kaohsiung Medical University (Approval number: IACUC-98146). Briefly, four male hairless mice, 6 weeks old BALB/c Nude mice (20–25 g), were obtained from the National Laboratory Animal Center, Taiwan. Mice were acclimatized and fed with a standard rat chow diet and water *ad libitum* in specific pathogen free laboratory for one week. All mice were received humane care in accordance to the “Guide for the Care and Use of Laboratory Animals” (National Academies Press, Washington, DC, USA, 1996). Norartocarpetin was dissolved in vehicle solution (propylene glycol 400:ethanol = 7:3 v ⁄ v) for external administration. The dorsal skin of mice was divided to four test sites (approximately 1.5 cm × 1.5 cm) for application and observation. The test period was three days and each mouse was topically treated once daily with 50 μl of vehicle solution, 0.02%, 0.1% and 0.2% norartocarpetin formulation in four test sites, respectively. The appearance of each application site was recorded at 24 h, 48 h and 72 h following external administration. The skin irritation of test sample, such as erythema or edema, was evaluated by the scoring system of Draize test, including (0): no erythema or no edema, (1): very slight erythema or edema, (2) well-defined erythema or edema, (3): moderate erythema or edema, (4): severe erythema or edema.

### Determination of cellular melanin content

Cellular melanin content was determined as described previously, with only slight modifications [24]. Briefly, 1 × 10^5^ B16F10 cells were seeded in 6-well plates and cultured at 37 overnight. B16F10 cells were then treated for 48 h with various concentrations of norartocarpetin (0.01–10 μM). Cells were then washed with PBS twice and lysed in 150 μl of 1 M NaOH. The lysate was heated at 95°C to solubilize the melanin, and then, 100 μl lysate was added in 1 well of a 96-well microplate. The plate was then quickly measured at 490 nm with a microplate spectrophotometer (BIOTEK, μQuant). In addition, we also tested the antimelanogenesis activity of norartocarpetin on α-MSH–induced melanogenesis. For this, B16F10 cells were treated with various concentrations of norartocarpetin for 24 h and then with 10 nM of α-MSH and incubated for 48 h. The determination of melanin content was performed as described above. All determinations were performed in triplicate.

### Determination of cellular tyrosinase activity

Cellular tyrosinase activity was measured as previously described [25], using a culture method similar to the melanin content assay. Briefly, the wells were treated with norartocarpetin in the presence or absence of 10 nM α-MSH for 48 h. Cells were then detached with trypsin-EDTA and centrifuged for 10 min at 12000 rpm in order to obtain cell pellets. The pellets lysed with 100 μl 1% Triton X-100 and 100 μl 0.1 mM PBS (pH 6.8) containing phenylmethylsulfonyl fluoride. The cell lysate was then frozen and thawed twice before being centrifuged for 10 min at 12000 rpm. The supernatant (80 μl) was added in a 96-well plate and mixed with 20 μl 0.2% l-DOPA. After incubation for 1 h, optical densities were measured at 475 nm using a microplate spectrophotometer (BIOTEK, μQuant). The inhibitory activity of the norartocarpetin-treated cells is presented as a percentage of the untreated cells.

### Analysis of melanogenesis protein expression by western blot

B16F10 cells were treated with 10 μM of norartocarpetin and in the presence or absence of α-MSH in a 6-well plate for 48 h. Cells were then collected and lysed in radioimmunoprecipitation assay (RIPA) buffer containing 50 mM Tris–HCl (pH 7.4), 150 mM NaCl, 1% NP-40, 0.5% sodium deoxycholate, 0.1% SDS, 2 mM phenylmethylsulfonyl fluoride, 1 mM sodium orthovanadate, and 2 g/ml each of aprotinin, leupeptin, and pepstatin. The lysates were centrifuged at 15,000 rpm for 10 min at 4°C before the supernatant was collected. The protein samples were then denatured and subjected to SDS–PAGE using a 12% running gel, before being transferred onto nitrocellulose membranes. Membranes were incubated with the following primary antibodies for 24 h: p-CREB, MITF, TYR, TRP1, TRP-2, p-ERK, p-p38, p-JNK, or GAPDH, and then incubated with anti-mouse or anti-rabbit horseradish peroxidase antibody for 1 h. The bands of protein expression were developed using ECL reagents and visualized using the Alphatec system. All determinations were performed in triplicate.

### Statistical analysis

All data were expressed as mean ± standard deviations of the indicated number of experiments. Statistical significance was determined using Student’s *t* test; a p value of <0.05 was considered significant.

## Results

### Norartocarpetin is a noncytotoxicity and non-skin irritation compound

**Figure 2:**
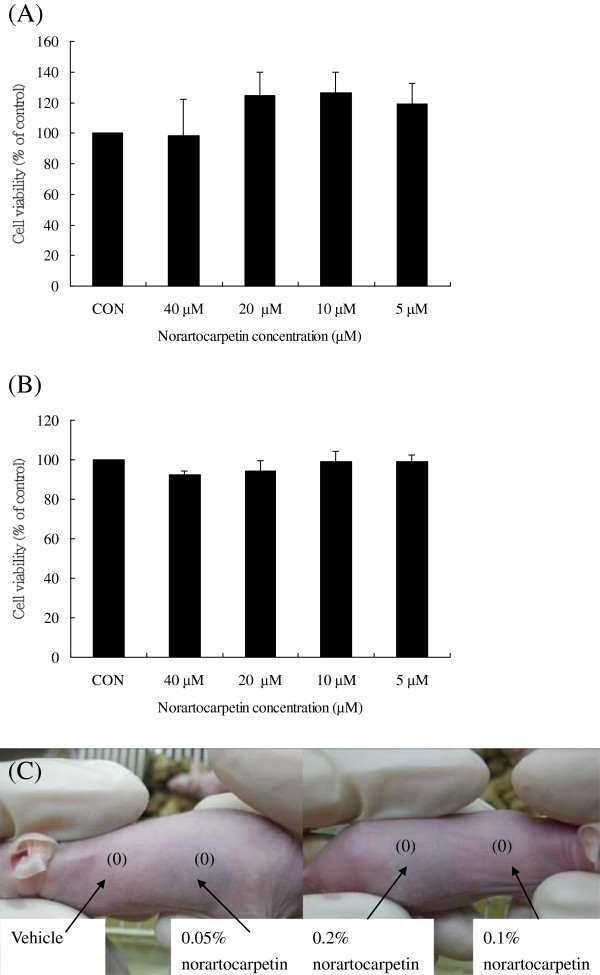
**The in vitro and in vivo safety of norartocarpetin.** Cell viability of norartocarpetin in B16F10 **(A)** and human dermal fibroblast cells **(B)** and skin irritation of norartocarpetin in nude mice **(C)**. Data are expressed as mean ± SD of 3 independent experiments. The different superscript letters indicate significant difference at p < 0.05 as analyzed by Student’s *t* test. *Significantly different from control. Scoring system of Draize test: (0): no erythema or no edema, (1): very slight erythema or edema, (2) well-defined erythema or edema, (3): moderate erythema or edema, (4): severe erythema or edema.

To be effective, active whitening compounds should decrease the melanin content in B16F10 melanoma cells with low cytotoxicity. To test the cytotoxicity of norartocarpetin, we treated B16F10 cells with various concentrations of norartocarpetin (5, 10, 20, and 40 μM) and determined cell viability using an MTT assay. As shown in Figure 2A, norartocarpetin concentrations ranging from 5 to 40 μM had no effect on cell viability after 48 h of treatment. In addition, Figure 2B indicated that the same concentration of norartocarpetin did not have any cytotoxic effects on human dermal fibroblasts. Moreover, the skin irritation of active ingredient is the major index of dermal safety in cosmetic application and therefore we conducted the Draize skin irritation test in BALB/c nude mice to confirm the skin irritation of norartocarpetin (Figure 2C). The three dose of norartocarpetin and vehicle solution were respectively scored 0 (no observable erythema or edema) according to the scoring system of Draize test. Based on the results from skin irritation test, no observable erythema or edema was found on the application site of BALB/c nude mice. The results indicated that norartocarpetin was found to have no skin irritation effect. Together, these results suggest that norartocarpetin might be a noncytotoxic and non-irritation compound for human medical and cosmetic applications. Therefore, we chose norartocarpetin concentrations of 1–10 μM to examine cellular melanin content and tyrosinase assay due to its non-cytotoxicity.

### Norartocarpetin effectively decreased cellular melanin content by inhibiting tyrosinase activity

**Figure 3:**
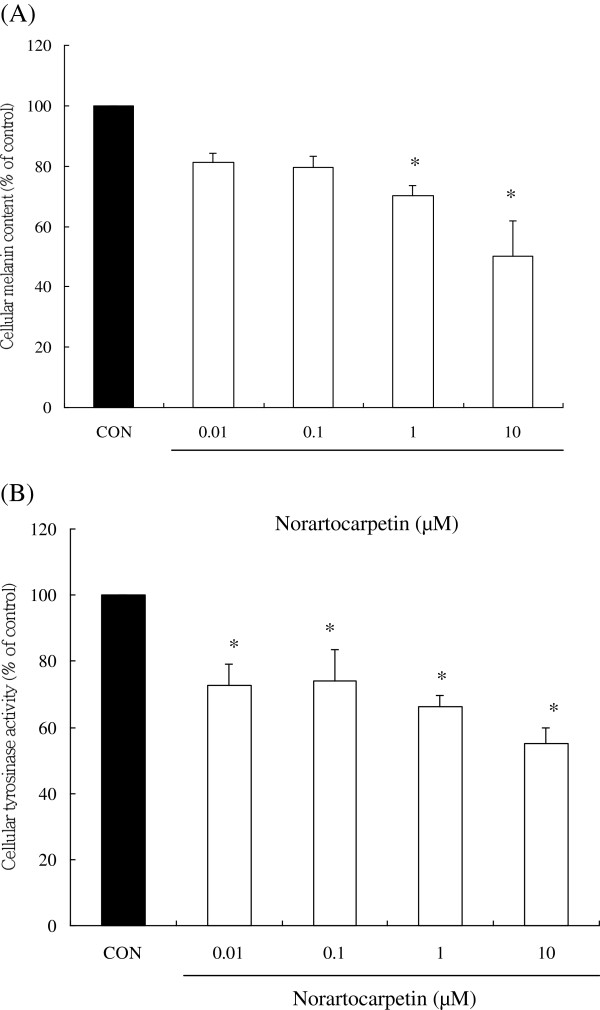
**Norartocarpetin decreases the melanogenesis in B16F10 cells.** Cellular melanin **(A)** and tyrosinase activity **(B)**. Data are expressed as mean ± SD of 3 independent experiments. The different superscript letters indicate significant difference at p < 0.05 as analyzed by Student’s *t* test. *Significantly different from control.

Tyrosinase is a rate-limiting enzyme in melanin biosynthesis, and enhancement of cellular melanin content plays an important role in melanogenesis [19-21]. Therefore, a good skin whitening agent would not only effectively inhibit cellular tyrosinase activity but also decrease melanin content. Figure 3A compares melanin content from B16F10 cells treated with DMSO (as control group) compared to those treated with norartocarpetin. Results show that the melanin contents of B16F10 cells treated with 0.01, 0.1, 1, and 10 μM of norartocarpetin have significantly decreased melanin content (P < 0.05), 81.08% ± 3.10%, 79.50% ± 3.89%, 70.13% ± 3.47%, and 50.06% ± 11.94%. Similar results were obtained for cellular tyrosinase activity assay (Figure 3B). B16F10 cells treated with 0.01, 0.1, 1, and 10 μM of norartocarpetin had significantly lower cellular tyrosinase activity (P < 0.05), i.e., 72.62% ± 6.48%, 73.96% ± 9.68%, 66.24% ± 3.42%, and 55.06% ± 4.81%, respectively. These results indicated that the treatment of B16F10 cells with various concentrations of norartocarpetin not only markedly decreased melanin content but also inhibited tyrosinase activity in a dose-dependent manner.

### Norartocarpetin inhibited tyrosinase activity by downregulating MITF and p-CREB protein

**Figure 4:**
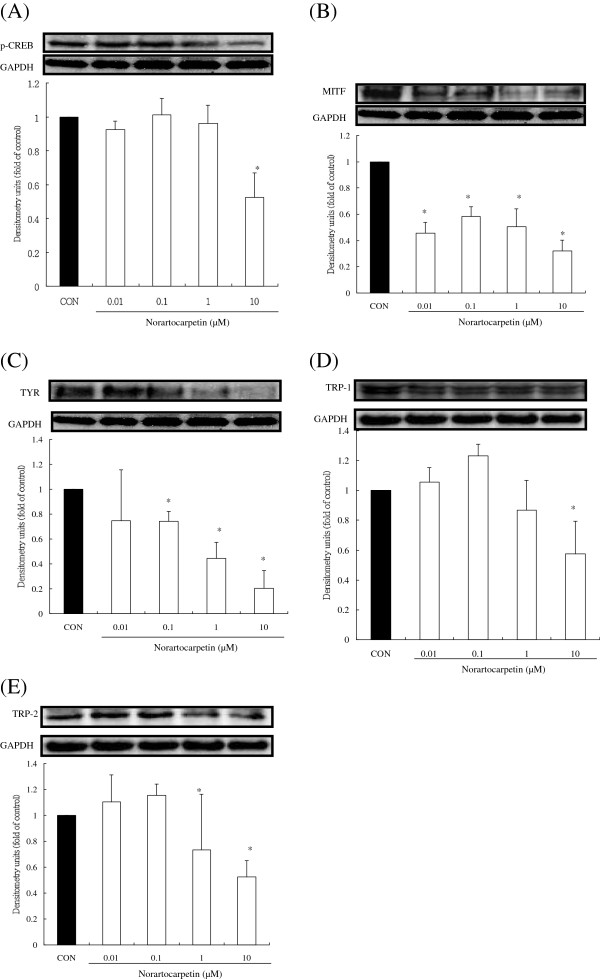
**Norartocarpetin downregulates the tryosinase-related protein expression. (A)** p-CREB, **(B)** MITF, **(C)** TYR, **(D)** TRP-1, **(E)** TRP-2. The different superscript letters indicate significant difference at p < 0.05 as analyzed by Student’s *t* test. *Significantly different from control.

It is well known that the synthesis of TYR, TRP-1, and TRP-2 is closely regulated through the activation of MITF and p-CREB protein [22-24]. Therefore, we used a western blot assay to determine the effect of various concentrations of norartocarpetin on the protein levels of MITF, p-CREB, TYR, TRP-1, and TRP-2. As shown in Figure 4, p-CREB and MITF are present in control melanoma cells that did not receive norartocarpetin treatment. Tyrosinase-related proteins (TYR, TRP-1, TRP-2) were also present in B16F10 cells that were not treated with norartocarpetin. These results indicated that B16F10 cells expressed tyrosinase-related proteins through the production of MITF and p-CREB protein. In B16F10 cells treated with norartocarpetin, we observed a dose-dependent decrease in p-CREB and MITF protein levels (P < 0.05). In turn, decreased TYR, TRP-1, and TRP-2 protein levels were also seen. This was particularly clear in the cells treated with 10 μM of norartocarpetin, which had obvious downregulation of p-CREB, MITF, TYR, TRP-1, and TRP-2. These results indicated that norartocarpetin inhibited tyrosinase-related protein levels, which is known to decrease melanin synthesis.

### Norartocarpetin can also inhibit α-MSH–induced melanogenesis

**Figure 5:**
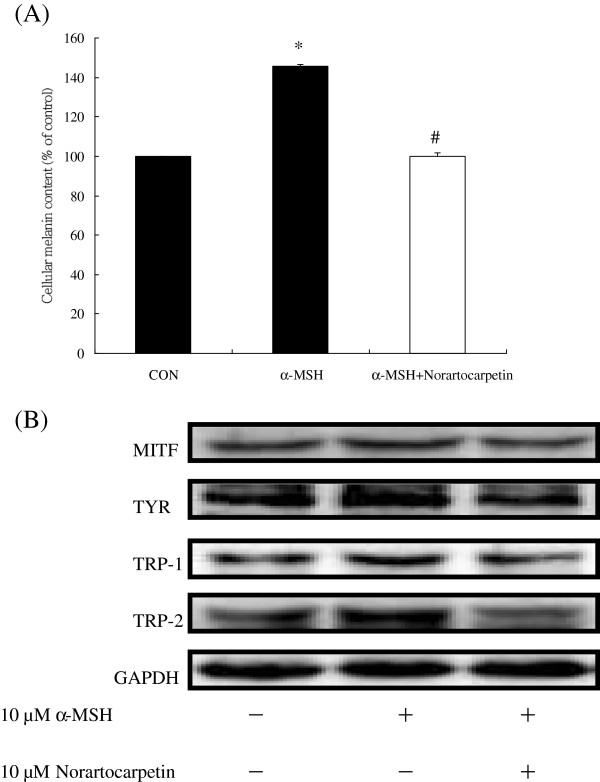
**Norartocarpetin decreases tyrosinase to inhibit α-MSH–induced melanogenesis.** Cellular melanin production **(A)** and downregulation of tyrosinase-related protein expression **(B)**. Data are expressed as mean ± SD of 3 independent experiments. The different superscript letters indicate significant difference at p < 0.05 as analyzed by Student’s *t* test. *Significantly different from control. #Significantly different from α-MSH–induced melanin production.

α-MSH is usually used to induce MITF protein overproduction, which leads to tyrosinase synthesis and melanin content enhancement, thereby causing melanogenesis. We therefore also treated B16F10 cells with 10 μM of norartocarpetin in an α-MSH–induced melanogenesis assay. Figure 5A indicates that α-MSH dramatically increased melanin content (145.83% ± 0.86%) when compared with the control. We found that treatment with 10 μM of norartocarpetin effectively decreased the melanin content (99.82% ± 2.07%) in α-MSH–induced B16F10 cells (P < 0.05). In addition, Figure 5B shows that 10 μM of norartocarpetin effectively decreased the MITF level and inhibited the TYR, TRP-1, and TRP-2 protein levels, which diminished the melanin content of α-MSH–induced B16F10 cells.

### Norartocarpetin downregulated MITF by activating phosphorylation of MAPKs

**Figure 6:**
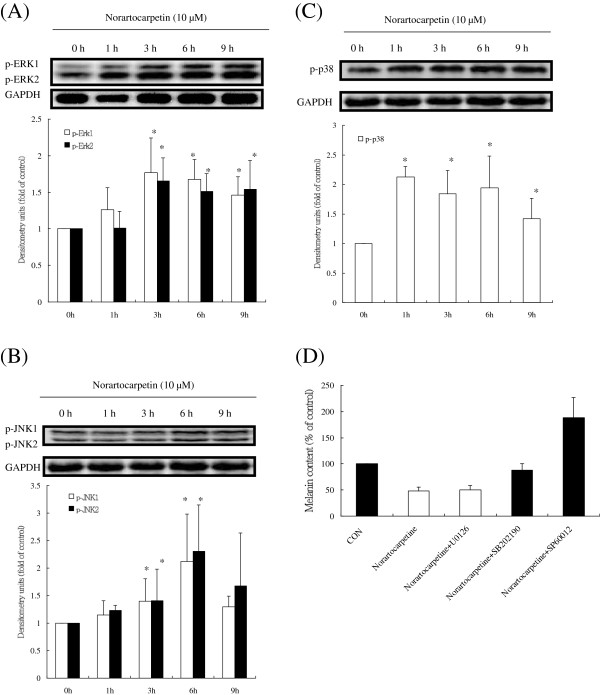
**Norartocarpetin inhibits tryosinase expression by activating phospho-MAPK. (A)** p-ERK, **(B)** p-JNK, **(C)** p-p38. **(D)** melanin content in cells treated for 24 h with MAPK inhibitors with or without 10 μM of norartocarpetin. The different superscript letters indicate significant difference at p < 0.05 as analyzed by Student’s *t* test. *Significantly different from that at 0 h.

**Figure 7:**
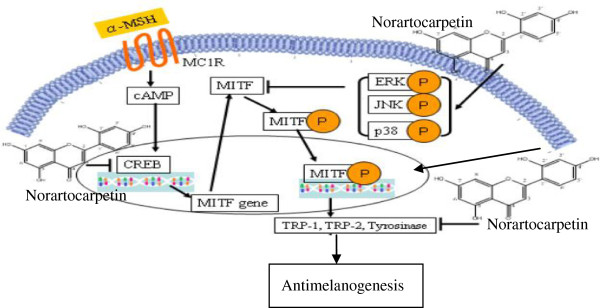
**The antimelanogenesis mechanism of norartocarpetin.** Norartocarpetin decreases cellular melanin production and tyrosinase activity by activating the phosphorylation of JNK and p38 and which results in reducing of MITF protein and p-CREB and inhibiting tyrosinase related protein synthesis including tyrosinase TRP-1 and TRP-2.

Previous studies have demonstrated that phosphorylation of MAPKs effectively degrades MITF, diminishes levels of tyrosinase proteins, and decreases melanin synthesis [25,26]. Therefore, we determined the effects of 10 μM of norartocarpetin on the levels of p-ERK, p-JNK, and p-p38 in a time course experiment. As shown in Figure 6, 10 μM of norartocarpetin enhanced ERK kinase, p38 kinase, and JNK kinase phosphorylation at 3, 6, and 1 h, respectively (P < 0.05). These data indicated that norartocarpetin may induce phosphorylation of three MAPKs and therefore, change the levels of MITF. The effects norartocarpetin on melanin synthesis were further tested by the addition 10 μM of U0126 (a selective inhibitor of MAPK/ERK), SB202190 (a selective inhibitor of p38), and SP600125 (a selective inhibitor of JNK). As shown in Figure 7, inhibition of p38 and JNK MAPKs by their selective inhibitors significantly reversed the antimelanogenesis activity of 10 μM of norartocarpetin (P < 0.05); however, there was no significant reverse effect on ERK inhibition. These results suggest that the antimelanogenesis activity of norartocarpetin depends on phosphorylation of the p38 and JNK pathways but not the ERK pathway.

## Discussion

In years past, hydroquinone, a skin whitening agent, is one of the most effective inhibitors of melanogenesis *in vitro* and *in vivo*; however, due to cytotoxic effects on melanocytes, it has a side effect of hypopigmentation, which can lead to vitiligo [26,27]. In addition, another common side effect of hydroquinone is skin peeling, redness, or skin sting. Based on these side effects, hydroquinone can not add into cosmetic for preventing skin darkness. Therefore, safety assessment is the first and major consideration in developing drug, health food and cosmetic. In cosmetic industry, the evaluation of cytotoxicity *in vitro* and skin irritation *in vivo* of active ingredient is the major index of dermal safety before drug and/or cosmetic product application. Many reports have recently indicated that skin whitening compounds shall be possessed non-cytotoxic effect for determining antimelanogenesis, such as quercetin [28], chrysin [29]. The present study performed cytotoxicity assays on B16F10 melanoma cells and normal human dermal fibroblasts to determine the cell viability of norartocarpetin. Our results demonstrated that norartocarpetin (5–40 μM) did not show significant cytotoxicity towards B16F10 cells (Figure 2A) or normal human dermal fibroblasts (Figure 2B). In addition, the dermal safety of active ingredient is the first consideration in cosmetic application, such as skin irritation. Our results demonstrated that norartocarpetin did not observe any erythema and edema in Draize test (Figure 2C). Based on these results, norartocarpetin is a non-cytotoxic and non-irritation compound and therefore the concentrations of norartocarpetin in the above range are used to determine the cellular melanin content, tyrosinase activity, and the molecular biological mechanism of antimelanogenesis.

There are several c-AMP activators used to induce the melanogenesis for developing skin whitening product, such as 3-Isobutyl-1-methylxanthine and α-MSH. It is well-known that α-MSH is a cAMP activator in human and vertebrate animal and which binds to melanocortin-1 receptor on melanocytes to stimulate the production of melanin and result in melanogenesis [18]. When taken in at stimulate dose, α-MSH becomes a potent melanogenesis agent, overproducing the cellular melanin content and tyrosinase activity. According that, the present study used α-MSH as melanogenesis activator to evaluate the antimelanogenesis activity of norartocarpetin. The results of the present study have demonstrated that 10 μM of norartocarpetin is effective as an antimelanogenesis agent since it decreases melanin content (Figure 3A) and tyrosinase activity (Figure 3B) in B16F10 cells. In addition, norartocarpetin can also decrease the α-MSH-activated melanogenesis effect that is usually used to stimulate melanin production in B16F10 cells (Figure 5A). Taken together, these results suggest that norartocarpetin is an effective tyrosinase inhibitor to decrease the melanin production in normal or α-MSH-stimulated conditions. Moreover, the overexpression of tyrosinase is the major rate-limiting step in melanin production. Many reports have demonstrated that CREB phosphorylation induces MITF protein enhancement, which in turn increases tyrosinase synthesis (TYR, TRP-1, and TRP-2) [19,22,23]. These tyrosinase-related proteins are the rate-limiting enzymes of melanogenesis and increase (1) the conversion of tyrosine to dopaquinone, (2) the rearrangement of DOPAchrome to 5,6-dihydroxy-indole-2-carboxylic acid, and (3) the overproduction and accumulation of melanin pigments in skin. Therefore, skin whitening ingredients such as paeonol [30] and curcumin [31] are effectively downregulated p-CREB and MITF proteins, as well as inhibited tyrosinase synthesis, so as to decrease melanin production. Our results demonstrate that norartocarpetin significantly downregulated the level of p-CREB, MITF, and its related proteins, including TYR, TRP1, and TRP2, in a dose-dependent manner. In addition (Figure 4), our data also demonstrated that α-MSH dramatically induced protein expression of MITF and increased the protein levels of TYR, TRP-1, and TRP-2. Our results also indicated that norartocarpetin treatment could diminish α-MSH–induced MITF protein levels, which resulted in reduced TYR, TRP-1, TRP-2 (Figure 5). In accordance with these findings, norartocarpetin treatment effectively decreased melanin production in B16F10 cells (Figure 3A) and/or α-MSH-induced B16F10 melanogenesis (Figure 5A).

On the other hand, previous studies have demonstrated that the MAPK signaling pathways (ERK, JNK, and p38) are major regulators of melanogenesis [20,24,30]. MAPK activation plays an important role in inducing MITF phosphorylation at serine-73, which leads to ubiquitination and subsequent MITF degradation, finally diminishing tyrosinase synthesis and melanin production [32]. Skin whitening agents that activate MAPK phosphorylation have been demonstrated to downregulate MITF protein expression and inhibit tyrosinase-related protein synthesis and melanin production [24,25,28-30]. Our study was firstly revealed that norartocarpetin can cause a significant increase in phosphorylation of ERK, JNK, and p38 MAPKs in a time-dependent manner. Activation of MAPKs downregulated MITF protein expression and further diminished tyrosinase (TRP-1 and TRP-2) synthesis, thereby inhibiting melanogenesis. Moreover, when we examined if the modulation of melanin production by norartocarpetin was regulated by MAPK signaling, we found that pretreatment with SB202190 (a selective inhibitor of p38) and SP600125 (a selective inhibitor of JNK) significantly reversed norartocarpetin-reduced melanin production. However, pretreatment with UO126 (a selective inhibitor of MAPK/ERK) did not reverse this. Thus, norartocarpetin-decreased melanin production was mediated through both the JNK and p38 pathways, consistent with reports indicating that activation of MAPKs inhibits melanin production in B16F10 melanoma cells.

On a different note, Alesiani et al. demonstrated that high concentrations of 5,7-dimethoxycoumarin (100–500 μM) showed the in vitro anticancer activity in melanoma cells through cell cycle arrest, differentiation induction and the compound can also inhibit the ERK 1/2 phosphorylation led to the B16 cell differentiation and melanogenesis process [33]. Gismondi et al. are also found that nimesulide, a non-steroidal anti-inflammatory drug, played as an antineoplastic agent to induce B16-F10 melanoma cell differentiation by enhancing the transglutaminase and tyrosinase activity and increase of melanin production [34]. In addition, Chen et al. revealed that α-MSH is a cancer stem cell-associated marker in melanoma through upregulating Wnt-1, β-catenin and MITF expression. Resveratrol at 15 μM could downregulate α-MSH stimulated cancer stem cell-associated molecules (Wnt-1, β-catenin and MITF expression) in melanoma B16 cells and finally decreased the cell proliferation, migration, and differentiation [35]. Moreover, Yajima et al. mentioned that MITF plays a “Two Faced” function role in melanoma development and progression. A low level of MITF expression promotes cell proliferation but a high level enhances cell differentiation through induction of cellular senescence and melanogenesis [36]. In the present data, norartocarpetin can downregulate the MITF expression and inhibit the melanogenesis and therefore it implicated that the anticancer activity of norartocarpetin is similar to resveratrol but the mechanism of norartocarpetin merits further investigation for cancer prevention application.

## Conclusion

The present study was firstly demonstrated that norartocarpetin is a safe compound due to noncytotoxicity and non-skin irritation. Norartocarpetin decreases cellular melanin production and tyrosinase activity by activating the phosphorylation of JNK and p38 and which results in reducing of MITF protein and p-CREB and inhibiting tyrosinase related protein synthesis including tyrosinase TRP-1 and TRP-2. Consequently, we suggest that as a result of its inhibitory effects on antimelanogenesis, norartocarpetin could be utilized as a skin whitening agent in the treatment of hyperpigmentation diseases and skin whitening cosmetics.

## Competing interests

The authors declare that they have no competing interests.

## Authors’ contributions

HHK designed the experiments and purified the norartocarpetin from *A. communis*. YTT, CJL, THY and CWL contributed bioactivity assay experiments and data analysis. MHY collected and identified the plants. CCL designed the experiments. FLY contributed the experiment designed, bioactivity assay experiments, data analysis and the animal model. All authors read and approved the final manuscript.

## Pre-publication history

The pre-publication history for this paper can be accessed here:

http://www.biomedcentral.com/1472-6882/13/348/prepub
